# Treating iPSC-Derived β Cells with an Anti-CD30 Antibody–Drug Conjugate Eliminates the Risk of Teratoma Development upon Transplantation

**DOI:** 10.3390/ijms23179699

**Published:** 2022-08-26

**Authors:** Silvia Pellegrini, Valentina Zamarian, Elisa Landi, Alessandro Cospito, Marta Tiffany Lombardo, Fabio Manenti, Antonio Citro, Marco Schiavo Lena, Lorenzo Piemonti, Valeria Sordi

**Affiliations:** 1 Diabetes Research Institute, IRCCS San Raffaele Hospital, Via Olgettina 60, 20132 Milan, Italy; 2 Department of Pathology, IRCCS San Raffaele Hospital, 20132 Milan, Italy; 3 Faculty of Medicine and Surgery, Vita-Salute San Raffaele University, 20132 Milan, Italy

**Keywords:** induced pluripotent stem cells, beta cells, cell therapy, type 1 diabetes, teratoma, CD30

## Abstract

Insulin-producing cells derived from induced pluripotent stem cells (iPSCs) are promising candidates for β cell replacement in type 1 diabetes. However, the risk of teratoma formation due to residual undifferentiated iPSCs contaminating the differentiated cells is still a critical concern for clinical application. Here, we hypothesized that pretreatment of iPSC-derived insulin-producing cells with an anti-CD30 antibody–drug conjugate could prevent in vivo teratoma formation by selectively killing residual undifferentiated cells. CD30 is expressed in all human iPSCs clones tested by flow cytometry (*n* = 7) but not in iPSC-derived β cells (iβs). Concordantly, anti-CD30 treatment in vitro for 24 h induced a dose-dependent cell death (up to 90%) in human iPSCs while it did not kill iβs nor had an impact on iβ identity and function, including capacity to secrete insulin in response to stimuli. In a model of teratoma assay associated with iβ transplantation, the pretreatment of cells with anti-CD30 for 24 h before the implantation into NOD-SCID mice completely eliminated teratoma development (0/10 vs. 8/8, *p* < 0.01). These findings suggest that short-term in vitro treatment with clinical-grade anti-CD30, targeting residual undifferentiated cells, eliminates the tumorigenicity of iPSC-derived β cells, potentially providing enhanced safety for iPSC-based β cell replacement therapy in clinical scenarios.

## 1. Introduction

Type 1 diabetes (T1D) is a disease resulting from the autoimmune-mediated destruction of insulin producing β cells of the pancreas. Despite significant advances in T1D management, exogenous insulin administration does not guarantee an optimal glycemic control and protection from long-term complications [1]. Allogeneic islet transplantation allows the reestablishment of glycemic control with the possibility of insulin independence, but this approach is severely limited by the scarcity of organ donors [2]. A new source of insulin-producing cells would significantly increase the possibility that cell therapy becomes a broad and standard therapy for diabetes treatment. Increasing advanced knowledge of the transition from pluripotent stem cells (PSCs) to insulin-secreting endocrine cells has led to significant improvements in protocols for differentiating β cells from PSC [3]. Today, PSC-derived β cells are appearing in the first pioneering clinical trials [4,5], and the issue of safety of the cellular product prior to implantation is therefore of crucial importance. In fact, PSCs can be differentiated into functional β cells in vitro [6,7,8,9], but not all the cells will reach complete differentiation, and a fraction of cells might remain undifferentiated [10]. We recently reported that the expression of pluripotency marker genes such as *OCT4* and *NANOG* is extremely low at the end of differentiation, but not completely cleared [11,12]. PSCs have the ability to differentiate into cells of three germinative lineages, and give rise to teratomas when transplanted in vivo [13]. Therefore, residual undifferentiated cells expose to a risk of teratoma formation [10,13]. Several groups have reported the formation of teratomatous tissue elements in grafts when unpurified PSC-derived pancreatic endoderm cells were infused in mice [14,15,16,17,18,19]. Many efforts to eliminate residual PSCs from differentiated cell cultures have been made, and essentially two different approaches have been developed: positive selection to purify target cells and negative selection to deplete undifferentiated cells. Regarding positive selection, antibody-mediated cell sorting strategies using surface markers CD24 [20], CD142 [19] or GP2 [21,22] were proposed, but these approaches hardly achieve complete purification and strongly affect cell number and viability. Regarding negative selection, more than one strategy has been suggested: the insertion of suicide genes through gene-editing strategies [23], the use of chemical inhibitors [24,25] and the use of selective killing by using antibodies [26,27]. In this regard, it was recently demonstrated that undifferentiated iPSCs may express the surface marker CD30 at high levels, while it is downregulated during mesoderm differentiation [28]. Brentuximab vedotin (BRE) is an antibody–drug conjugate (anti-CD30 antibody linked to the antitubulin agent monomethyl auristatin E [29]) able to kill CD30-positive cells. BRE is already approved for clinical use in the treatment of certain CD30-expressing lymphomas [30], and this makes its use attractive for the development of clinical-grade methods to eliminate residual undifferentiated CD30^+^ iPSCs [31]. Here, we test the possibility of using BRE, after iPSC endoderm differentiation into insulin-producing cells, to eliminate the risk of teratoma development by selectively killing residual undifferentiated CD30^+^ cells.

## 2. Results

### 2.1. CD30 Expression in iPSCs during Endoderm Differentiation

**Figure 1:**
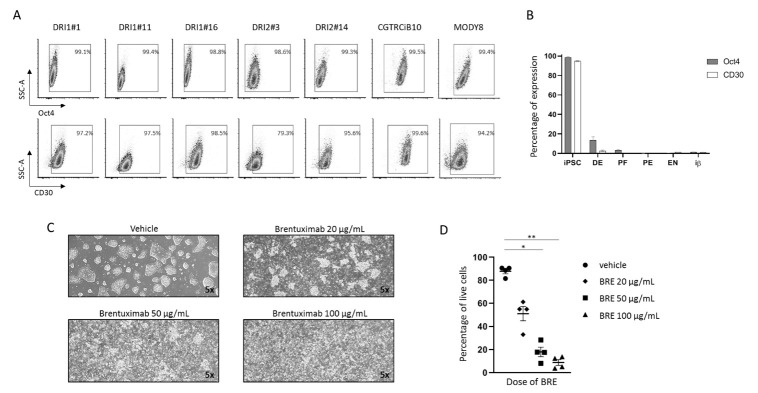
CD30 expression in iPSCs during differentiation into β cells and effect of brentuximab vedotin administration on undifferentiated iPSCs. (**A**). Representative plots of the percentage of cells positive for Oct4 (upper plots) and CD30 (lower plots) in flow cytometry in seven undifferentiated iPSC clones. Gate delimitates positive cells. (**B**). Histogram plot of the percentages of Oct4-and CD30-positive cells during the steps of differentiation (iPSC, DE, PF, PE, ENs and iβs) of MODY8 iPSC in four independent differentiation experiments. Results are expressed as mean ± SEM. (**C**). MODY8 iPSC morphology after 24 h treatment with vehicle or 20, 50, 100 µg/mL of BRE. Magnification 5×. (**D**). Percentage of MODY8 iPSC live cells in flow cytometry in the tested conditions. Each dot represents one experiment * = *p* < 0.05, ** = *p* < 0.01.

The presence of the protein CD30, a cell membrane protein of the tumor necrosis factor receptor family and tumor marker, was tested on six different iPSC lines (CGTRCiB10, DRI1#1, DRI1#11, DRI1#16, DRI2#3, DRI2#14) reprogrammed from healthy controls, and one from a patient with monogenic diabetes (MODY8). Virtually all the iPSCs at the stage of pluripotency were positive for CD30 in flow cytometry (95.33 ± 0.8% in CGTRCiB10, 94.7 ± 0.5% in MODY8, 99.1 ± 0.3% in DRI1 clones, 99.3 ± 0.7% in DRI2 clones, *n* ≥ 3, Figure 1A). iPSCs were differentiated into β cells (iβs) as previously described [11,12]. The analysis of the different steps of differentiation from iPSCs to DE, PF, PE, ENs and iβs revealed that CD30 expression was drastically reduced at DE and PF stages and almost disappeared at the final stages of PE, ENs and iβs. The presence of CD30 coincided with that of the Oct4 pluripotency marker, both in the undifferentiated iPSCs and along the different maturation steps (Figure 1B and Supplementary Figure S1A). A small contaminant of CD30-positive cells (0.59 ± 0.05% in CGTRCiB10, 0.94 ± 0.16% in MODY8, *n* = 3) was present in the terminally differentiated cells. Human pancreas, isolated islets from organ donors and the immortalized human β cell line were negative for CD30 expression (Supplementary Figure S1B).

### 2.2. Brentuximab Vedotin Effect on Undifferentiated iPSCs

iPSCs were treated with increasing concentrations of the monoclonal antibody anti-CD30 BRE. The drug had a cytotoxic effect already at the lowest dose, which increased dose-dependently. Cell morphology clearly revealed massive cell death after 24 h of treatment (Figure 1C), and the percentage of live cells in flow cytometry decreased from 87.9 ± 2.2% to 51.2 ± 6.2%, 18 ± 4.1% (*p* < 0.05 vs. vehicle) and 8.9 ± 2.5% (*p* < 0.01 vs. vehicle) at 0, 20, 50 and 100 µg/mL of BRE, respectively (*n* = 4, Figure 1D). 

### 2.3. Brentuximab Vedotin Effect on iPSC-Derived β Cells

**Figure 2:**
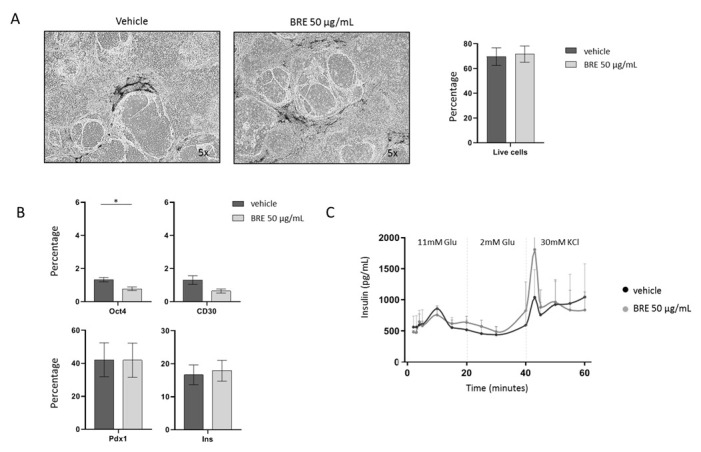
Effect of brentuximab vedotin on MODY8 iPSC-derived β cells. (**A**). Morphology of iβs treated with vehicle or 50 µg/mL BRE, magnification 5×, and percentage of live cells in flow cytometry (*n* = 5). (**B**). Percentage of Oct4-, CD30-, Pdx1- and Insulin-positive cells gated on live cells in control (dark gray) and BRE (light gray)-treated cells (*n* = 5, * = *p* < 0.05). (**C**). Insulin secretion profile in vehicle (black line) and BRE (gray line)-treated iβs in a dynamic perifusion assay (*n* = 3).

iβs were treated with vehicle or 50 µg/mL BRE for 24 h. Treatment with BRE did not affect cell viability (Figure 2A). We observed a reduction in the residual percentage of cells positive for Oct4 or CD30, while the percentage of cells positive for insulin and/or Pdx1 was not influenced, which indicates that BRE is not harmful to differentiated cells (Figure 2B). More importantly, the capacity of iβs to respond to a glucose stimulus in a dynamic perifusion assay was not significantly affected by the treatment with BRE (Figure 2C). In two batches of iβs, cells were cultured for 7 days after 24 h of BRE treatment and washed out: cell vitality and the Pdx1/Insulin-positive cell percentage were not different from control iβs. These data suggest that BRE has a selective action on CD30^+^ residual undifferentiated cells and does not affect endodermal-differentiated insulin-producing cells.

### 2.4. Brentuximab Vedotin Treatment of iβs Prior Transplantation Prevents Teratoma Formation

**Figure 3:**
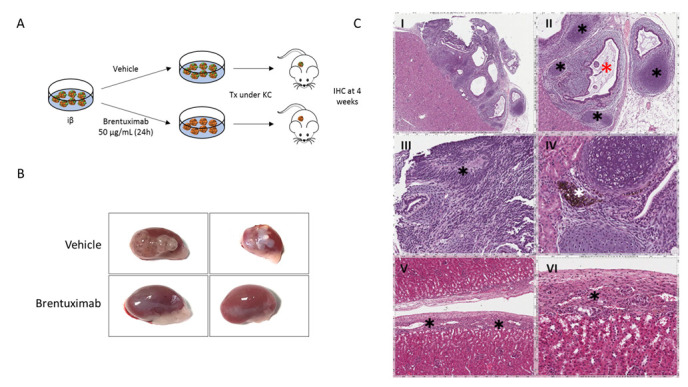
Effect of Brentuximab vedotin pretreatment on iβs 4 weeks after transplantation. (**A**). Schematic representation of iβ pretreatment with vehicle or BRE and transplantation under the kidney capsule. (**B**). Gross pathology of kidneys explanted 4 weeks after transplant of iβs treated with vehicle (upper images) or BRE (lower images). (**C**). Hematoxilin and Eosin stain of grafts explanted 4 weeks after iβ transplantation: mice not pretreated with BRE developed teratoma at the graft site (I). Islands of condroid tissue (black asterisks, panel II), glandular structures (red asterisk, panel II) immersed in an immature mesenchymal tissue, rosette-forming neuroepithelium (black asterisk, panel III) or pigmented retinal-type epithelium (white asterisk, panel IV) were observed. In mice pretreated with BRE, no tumor developed. Endocrine islet-type cells can be seen in the renal capsule (V and VI).

Among the available iPSC clones, iβs derived from MODY8 showed an ability to develop teratoma in nearly 100% of the mice after transplantation and were chosen for the in vivo BRE efficacy studies. This characteristic allowed us not to mix pluripotent cells with terminally differentiated cells in order to verify the effect of BRE on teratoma formation, like in other reported experimental approaches [27,28,32]. Two million iβs, pretreated with vehicle or 50 µg/mL BRE for 24 h, were transplanted under the kidney capsule of NOD/SCID mice and four weeks after transplantation, grafts were explanted and the presence of teratoma evaluated by gross pathology and histological analysis (Figure 3A). BRE pretreatment abolished the formation of teratoma in the graft. In fact, 0 out of 10 mice transplanted with cells pretreated with BRE developed neoplastic growth, and in all the explanted kidneys it was possible to identify the implant area with the transplanted cells without evidence of any renal parenchyma invasion (Figure 3B,C). On the other hand, eight out of eight mice transplanted with cells pretreated with vehicle (*p* < 0.01 vs. BRE, Fisher’s exact test) developed a kidney tumoral mass (Figure 3B). The masses, confined to the graft implant area, were classified as teratoma by the independent analysis of an expert pathologist (Figure 3C).

## 3. Discussion

In this study, we investigated the potential of brentuximab vedotin to abolish the tumorigenicity of human-iPSC-derived β cells, providing enhanced safety for iPSC-based β cell replacement therapy in clinical scenarios. This work reinforces what has already been described by Sougawa et al. for the selection of cardiomyocytes derived from stem cells [28] and extends the potential use of BRE to increase the safety profile of cells to be transplanted in the cell therapy of diabetes. BRE is an anti-CD30 chimeric antibody conjugated to the antimicrotubule agent monomethyl auristatin E (MMAE). MMAE induces cell cycle arrest in the G2/M phase followed by apoptosis, leading to cytotoxic effects [31,33]. Since BRE targets CD30-positive cells and immature iPSCs express CD30, BRE could selectively kill undifferentiated iPSCs. We observed that CD30 was highly expressed on several lines of iPSC, reprogrammed from healthy and diabetic donors, while it was not present on pancreatic-differentiated cells such as donor pancreas, human islets and the immortalized β cell line. Furthermore, the expression level of CD30 on iPSCs rapidly diminished with ongoing endodermal differentiation into β cells. These data suggest that the CD30 antibody–cytotoxic drug conjugate BRE has the potential to remove residual undifferentiated iPSCs by β cell preparation before transplantation. Our experiments confirmed that treatment with BRE in vitro dose-dependently induced death in iPSCs and that the pretreatment of iβs for 24 h led to prevention of teratoma formation in NOD-SCID mice at one month after transplantation. It is not possible to exclude the possibility that longer follow-ups may lead to the growth of teratomatous cells even if such a strong effect at the 4-week time point is an indicator of a severe outcome on the survival and tumor-forming capacity of the grafted cells. Other approaches aimed at generating cellular products with high safety, devoid of potentially tumorigenic pluripotent cells, have been tested. Cell-sorting strategies based on the expression of specific surface markers for pancreatic progenitor cells or for mature β cells are under investigation. The most promising markers tested include SEZ6L2 (seizure-related 6 homologue-like 2), LRP11 (low-density lipoprotein-receptor-related protein 11), DISP2 (dispatched homologue 2 Drosophila) and SLC30A8 (solute carrier family 30 zinc transporter member 8) [34], the pancreatic secretory granule membrane major glycoprotein 2 (GP2) [21,22], the platelet tissue factor CD142 [19] and α1 integrin CD49a [35]. Cell sorting, however, exerts a strong mechanical stress that can induce massive cell death, and the balance between yield and purity of the sorted cell population must be carefully evaluated. Another option under investigation is the use of chemical ablation of pluripotent cells. Benvenisty et al. performed a high-throughput screen of over 52,000 small molecules and identified 15 pluripotent cell-specific inhibitors, named PluriSIns, which could eliminate PSCs while sparing a large array of progenitors and differentiated cells [24]. The limit of PluriSIn and other similar compounds mainly regards their real selectivity and their potential toxicity for the differentiated cells. Finally, another method for selecting the desired cells and getting rid of potentially tumorigenic ones is gene editing. Through genetic manipulation, in fact, it is possible to select certain cell types following genetic labeling approaches. These include the insertion of a reporter gene (eGFP) under the transcriptional control of a tissue-specific promoter [36], the knockout of tumor progression genes [37] or the introduction of a suicide gene into a genetic locus highly specifically expressed in pluripotent stem cells but not in their differentiated derivatives [38]. However, although genetic manipulation is an increasingly common strategy within the reach of every lab, it is not free from drawbacks such as limited efficiency, the need for single-cell dissociation and the danger of off-target DNA damages. Finally, all these strategies, proposed to solve the problem of tumorigenicity in PSCs, should take the path to clinical approval. An advantage of our approach is that BRE is a drug that has been already approved for clinical application by the Food and Drug Administration and European Medicines Agency, making this strategy easier to translate into clinical practice.

In conclusion, β cell replacement therapy would require billions of iPSC-derived β cells to improve glycemic control, emphasizing the necessity of developing methods that promise safety and efficacy. Although various approaches, such as cell sorting strategies, chemical inhibitors and introduction of suicide genes, have been reported to remove tumorigenic cells, all of them have some limitations in terms of poor efficacy, low cell recovery or high cost. Treatment with BRE induces cell death in immature iPSCs and reduces tumorigenicity of iPSC-derived β cells, suggesting that this strategy is feasible and immediately able to increase the safety of clinical applications of iPSC-based cell therapy for diabetes.

## 4. Materials and Methods

### 4.1. Human Islets and EndoC-βH1

Human pancreatic islet (HI) preparations were isolated from heart-beating cadaveric organ donors as previously described [39] in the Pancreatic Islet Processing Unit of the Diabetes Research Institute (DRI) at the San Raffaele Hospital in Milan, Italy. The use of human specimens (islet preparations discarded from clinical use) was approved by the Institutional Review Board under the “European Consortium for Islet Transplantation (ECIT) human islet distribution program” supported by the Juvenile Diabetes Research Foundation (JDRF) (3-RSC-2016-160-I-X) [40]. The genetically engineered human pancreatic β cell line EndoC-βH1 [41] was grown in DMEM-low glucose (1 g/L) (Gibco, New York, NY, USA), 2% BSA (Sigma), 50 μM 2-mercaptoethanol (Sigma, St. Louis, MO, USA), 10 mM nicotinamide (Sigma), 5.5 μg/mL transferrin (Gibco), 6.7 ng/mL sodium selenite (Sigma), 1% Pen/Strep.

### 4.2. Induced Pluripotent Stem Cells

Human iPSC cell line MODY8-iPSC was obtained from somatic reprogramming of patients’ skin fibroblasts with Sendai virus delivery of reprogramming factors, as recently described [12]. Human iPSC cell line CGTRCiB10 was obtained from Cell and Gene Therapy Catapult, London, UK. Human iPSC cell lines DRI1 (clones 1, 11 and 16) and DRI2 (clones 3 and 14) were obtained from somatic reprogramming of blood cells of two healthy subjects with Sendai virus delivery of reprogramming factors. Cells were cultured in six-well plates (Corning Incorporated, Costar) with 0.5 μg/cm² Vitronectin Recombinant human protein (ThermoScientific) in Essential 8 Basal Medium (Gibco) supplemented with 1% Pen/Strep (Lonza) and cultured at 37°C and 5% CO_2_. Cells were passed every 3–4 days using 0.5 mM EDTA (ThermoScientific). Cells were imaged using an EVOS microscope (ThermoScientific). 

### 4.3. Differentiation Protocol and Brentuximab Vedotin Treatment

IPSCs were differentiated into endocrine pancreatic cells following our recently described 25-day protocol [11,12]. During differentiation, medium was changed every day and cells were maintained in adhesion in 6-well plates (Costar, Washington, DC, USA) and cultured at 37 °C and 5% CO_2_. Brentuximab vedotin (BRE, Adcetris^®^, Takeda, Tokyo City, Japan, gently provided by leftovers of San Raffaele Hospital pharmacy) was used on iPSCs at three different concentrations, 20, 50 and 100 μg/mL, for 24 h and on iβs at 50 μg/mL for 24 h. 

### 4.4. Cytofluorimetric Analysis

Cytofluorimetric analysis was performed on iPSCs at specific stages of differentiation: undifferentiated cells, iPSCs; definitive endoderm, DE; posterior foregut, PF; pancreatic endoderm, PE; endocrine cells, ENs; β-like cells, iβs. At a specific time points, cells were detached using 1x Trypsin-EDTA (Lonza, Basel, Switzerland) and stained with Live/Dead Fixable Violet stain kit (ThermoScientific, Waltham, MA, USA) to exclude dead cells from the analysis. For intracellular staining, cells were fixed with Cytofix/Cytoperm (Becton Dickinson, BD, Franklin Lakes, NJ, USA) and then permeabilized with BD PhosflowTM Perm Buffer III (BD). Cells were stained using the following monoclonal antibodies: Oct4: 40/Oct3 Alexa-Fluor647^®^ Mouse anti-OCT3/4; CD30: Ber-H8 PE Mouse Anti Human-CD30; Pdx1: 658A5 Alexa-Fluor488^®^ Mouse Anti-PDX-1; Insulin: T56-706 Alexa-Fluor647^®^ Mouse anti-Insulin (all from BD, Biosciences). Analysis was carried out on a FACS Canto cytometer (BD) using FACS Diva software. The results were analyzed with FCS Express 7 Flow Research Edition (FCS Express™ 7.12.0005, De Novo Software, Pasadena, CA, USA). 

### 4.5. Dynamic Insulin Secretion

At the iβ step, an automated perifusion system (BioRep^®^ Perifusion V2.0.0, Biorep Technologies Inc., Miami Lakes, FL, USA) was used to stimulate dynamically insulin secretion. The day before the test, cells were transferred in an ultra-low attachment plate (Costar) to allow cluster formation in suspension and BRE added at 50 μg/mL for 24 h. One thousand clusters (corresponding to approximately one million cells) were loaded in perifusion chambers and immobilized in Bio-Gel P-4 Gel (BioRad, Hercules, CA, USA). A peristaltic pump allows for the perfusion of cells with different solutions with a constant flow rate and a steady temperature of 37 °C, as previously described [11,12]. Insulin released during dynamic perifusion was measured with the ELISA kit for human insulin (Mercodia, Winston Salem, NC, USA), following the manufacturer’s instructions. The plates were analyzed using an ELISA reader (MicroPlate Reader, Model 680, BioRad).

### 4.6. Transplantation of iPSC-Derived iβs 

The day before transplant, iβs were transferred to an ultra-low attachment plate to allow cluster formation in suspension, treated for 24 h with 50 μg/mL BRE or with PBS and then 2 million iβs were transplanted as clusters under the kidney capsule of NOD/SCID mice (male, 8 weeks old, Charles River). After four weeks, during which the weight and health conditions of the mice were monitored, mice were sacrificed and the transplanted kidneys explanted for further analysis. The local animal ethics committee approved all experiments (IACUC n 686/2017-PR).

### 4.7. Immunohistochemistry Analysis

Explanted mouse kidneys and samples from human pancreas were fixed in 10% formalin (Sigma), paraffin-embedded and processed for immunohistochemical analysis. Kidney sections were marked with hematoxylin–eosin (Bio-Optica, Milan, Italy), and pancreas sections from organ donor were stained with monoclonal antibody anti-CD30 (Clone Ber-H2, Dako, Agilent Technologies, Santa Clara, CA, USA), and counterstained with hematoxylin. Stained sections were analyzed with color camera, which allows for scanning and digitizing sections with vertical multiple scans at 20x magnification (AperioScanscope, Leica, Wetzlar, Germany).

### 4.8. Statistical Analysis

Nonparametric test (Kruskal–Wallis with Dunn’s multiple comparison test or Mann–Whitney) was used to compare groups, and a 2-tailed *p* value less than 0.05 or 0.01 was considered significant. Data are reported as mean ± SEM. Analysis of data was performed using the Prism software (GraphPad Prism 5).

## Data Availability

The data that support the findings of this study are available on request from the corresponding author.

## Supplementary Materials

The supporting information can be downloaded at: https://www.mdpi.com/article/10.3390/ijms23179699/s1.

## References

[1] Piemonti L. (2021). Felix dies natalis, insulin… ceterum autem censeo “beta is better”. Acta Diabetol..

[2] Shapiro A.M.J., Pokrywczynska M., Ricordi C. (2017). Clinical pancreatic islet transplantation. Nat. Rev. Endocrinol..

[3] Melton D. (2021). The promise of stem cell-derived islet replacement therapy. Diabetologia.

[4] Ramzy A., Thompson D.M., Ward-Hartstonge K.A., Ivison S., Cook L., Garcia R.V., Loyal J., Kim P.T.W., Warnock G.L., Levings M.K. (2021). Implanted pluripotent stem-cell-derived pancreatic endoderm cells secrete glucose-responsive C-peptide in patients with type 1 diabetes. Cell Stem Cell.

[5] Shapiro A.J., Thompson D., Donner T.W., Bellin M.D., Hsueh W., Pettus J., Wilensky J., Daniels M., Wang R.M., Brandon E.P. (2021). Insulin expression and C-peptide in type 1 diabetes subjects implanted with stem cell-derived pancreatic endoderm cells in an encapsulation device. Cell Rep. Med..

[6] Pagliuca F.W., Millman J.R., Gürtler M., Segel M., Van Dervort A., Ryu J.H., Peterson Q.P., Greiner D., Melton D.A. (2014). Generation of functional human pancreatic β cells in vitro. Cell.

[7] Rezania A., Bruin J.E., Arora P., Rubin A., Batushansky I., Asadi A., O’Dwyer S., Quiskamp N., Mojibian M., Albrecht T. (2014). Reversal of diabetes with insulin-producing cells derived in vitro from human pluripotent stem cells. Nat. Biotechnol..

[8] Pellegrini S., Manenti F., Chimienti R., Nano R., Ottoboni L., Ruffini F., Martino G., Ravassard P., Piemonti L., Sordi V. (2018). Differentiation of Sendai Virus-Reprogrammed iPSC into β Cells, Compared with Human Pancreatic Islets and Immortalized β Cell Line. Cell Transplant..

[9] Millman J.R., Xie C., Van Dervort A., Gürtler M., Pagliuca F.W., Melton D.A. (2016). Generation of stem cell-derived b-cells from patients with type 1 diabetes. Nat. Commun..

[10] Pellegrini S., Cantarelli E., Sordi V., Nano R., Piemonti L. (2016). The state of the art of islet transplantation and cell therapy in type 1 diabetes. Acta Diabetol..

[11] Pellegrini S., Chimienti R., Scotti G.M., Giannese F., Lazarevic D., Manenti F., Poggi G., Lombardo M.T., Cospito A., Nano R. (2021). Transcriptional dynamics of induced pluripotent stem cell differentiation into β cells reveals full endodermal commitment and homology with human islets. Cytotherapy.

[12] Pellegrini S., Pipitone G.B., Cospito A., Manenti F., Poggi G., Lombardo M.T., Nano R., Martino G., Ferrari M., Carrera P. (2021). Generation of β cells from iPSC of a MODY8 patient with a novel mutation in the carboxyl ester lipase (CEL) gene. J Clin. Endocrinol. Metab..

[13] Ben-David U., Benvenisty N. (2011). The tumorigenicity of human embryonic and induced pluripotent stem cells. Nat. Rev. Cancer.

[14] El Khatib M.M., Ohmine S., Jacobus E.J., Tonne J.M., Morsy S.G., Holditch S.J., Schreiber C.A., Uetsuka K., Fusaki N., Wigle D.A. (2016). Tumor-Free Transplantation of Patient-Derived Induced Pluripotent Stem Cell Progeny for Customized Islet Regeneration. Stem Cells Transl. Med..

[15] Kroon E., Martinson L.A., Kadoya K., Bang A.G., Kelly O.G., Eliazer S., Young H., Richardson M., Smart N.G., Cunningham J. (2008). Pancreatic endoderm derived from human embryonic stem cells generates glucose-responsive insulin-secreting cells in vivo. Nat. Biotechnol..

[16] Rezania A., Bruin J.E., Riedel M.J., Mojibian M., Asadi A., Xu J., Gauvin R., Narayan K., Karanu F., O’Neil J.J. (2012). Maturation of human embryonic stem cell–derived pancreatic progenitors into functional islets capable of treating pre-existing diabetes in mice. Diabetes.

[17] Parent A.V., Ashe S., Nair G.G., Li M.L., Chavez J., Liu J.S., Zhong Y., Streeter P.R., Hebrok M. (2022). Development of a scalable method to isolate subsets of stem cell-derived pancreatic islet cells. Stem Cell Rep..

[18] Fujikawa T., Oh S.H., Pi L., Hatch H.M., Shupe T., Petersen B.E. (2005). Teratoma formation leads to failure of treatment for type I diabetes using embryonic stem cell-derived insulin-producing cells. Am. J. Pathol..

[19] Kelly O.G., Chan M.Y., Martinson L.A., Kadoya K., Ostertag T.M., Ross K.G., Richardson M., Carpenter M.K., D’Amour K.A., Kroon E. (2011). Cell-surface markers for the isolation of pancreatic cell types derived from human embryonic stem cells. Nat. Biotechnol..

[20] Jiang W., Sui X., Zhang D., Liu M., Ding M., Shi Y., Deng H. (2011). CD24: A novel surface marker for PDX1-positive pancreatic progenitors derived from human embryonic stem cells. Stem Cells.

[21] Cogger K.F., Sinha A., Sarangi F., McGaugh E.C., Saunders D., Dorrell C., Mejia-Guerrero S., Aghazadeh Y., Rourke J.L., Screaton R.A. (2017). Glycoprotein 2 is a specific cell surface marker of human pancreatic progenitors. Nat. Commun..

[22] Aghazadeh Y., Sarangi F., Poon F., Nkennor B., McGaugh E.C., Nunes S.S., Nostro M.C. (2022). GP2-enriched pancreatic progenitors give rise to functional beta cells in vivo and eliminate the risk of teratoma formation. Stem Cell Rep..

[23] Schuldiner M., Itskovitz-Eldor J., Benvenisty N. (2003). Selective ablation of human embryonic stem cells expressing a “suicide” gene. Stem Cells.

[24] Ben-David U., Gan Q.F., Golan-Lev T., Arora P., Yanuka O., Oren Y.S., Leikin-Frenkel A., Graf M., Garippa R., Boehringer M. (2013). Selective elimination of human pluripotent stem cells by an oleate synthesis inhibitor discovered in a high-throughput screen. Cell Stem Cell.

[25] Lee M.O., Moon S.H., Jeong H.C., Yi J.Y., Lee T.H., Shim S.H., Rhee Y.H., Lee S.H., Oh S.J., Lee M.Y. (2013). Inhibition of pluripotent stem cell-derived teratoma formation by small molecules. Proc. Natl. Acad. Sci. USA.

[26] Ben-David U., Nudel N., Benvenisty N. (2013). Immunologic and chemical targeting of the tight-junction protein Claudin-6 eliminates tumorigenic human pluripotent stem cells. Nat. Commun..

[27] Tang C., Lee A.S., Volkmer J.P., Sahoo D., Nag D., Mosley A.R., Inlay M.A., Ardehali R., Chavez S.L., Pera R.R. (2011). An antibody against SSEA-5 glycan on human pluripotent stem cells enables removal of teratoma-forming cells. Nat. Biotechnol..

[28] Sougawa N., Miyagawa S., Fukushima S., Kawamura A., Yokoyama J., Ito E., Harada A., Okimoto K., Mochizuki-Oda N., Saito A. (2018). Immunologic targeting of CD30 eliminates tumourigenic human pluripotent stem cells, allowing safer clinical application of hiPSC-based cell therapy. Sci. Rep..

[29] Okeley N.M., Miyamoto J.B., Zhang X., Sanderson R.J., Benjamin D.R., Sievers E.L., Senter P.D., Alley A.C. (2010). Intracellular activation of SGN-35, a potent anti-CD30 antibody-drug conjugate. Clin. Cancer Res..

[30] Deng C., Pan B., O’Connor O.A. (2013). Brentuximab Vedotin. Clin. Cancer Res..

[31] Francisco J.A., Cerveny C.G., Meyer D.L., Mixan B.J., Klussman K., Chace D.F., Rejniak S.X., Gordon K.A., DeBlanc R., Toki B.E. (2003). cAC10-vcMMAE, an anti-CD30-monomethyl auristatin E conjugate with potent and selective antitumor activity. Blood.

[32] Hentze H., Soong P.L., Wang S.T., Phillips B.W., Putti T.C., Dunn N.R. (2009). Teratoma formation by human embryonic stem cells: Evaluation of essential parameters for future safety studies. Stem Cell Res..

[33] Sanderson R.J., Hering M.A., James S.F., Sun M.M., Doronina S.O., Siadak A.W., Senter P.D., Wahl A.F. (2005). In vivo drug-linker stability of an anti-CD30 dipeptide-linked auristatin immunoconjugate. Clin. Cancer Res..

[34] Hald J., Galbo T., Rescan C., Radzikowski L., Sprinkel A.E., Heimberg H., Poh Y.C., Sintov E., Gürtler M., Pagliuca F.W. (2012). Pancreatic islet and progenitor cell surface markers with cell sorting potential. Diabetologia.

[35] Veres A., Faust A.L., Bushnell H.L., Engquist E.N., Kenty J.H.R., Harb G., Poh Y.C., Sintov E., Gürtler M., Pagliuca F.W. (2019). Charting cellular identity during human in vitro β-cell differentiation. Nature.

[36] Eiges R., Schuldiner M., Drukker M., Yanuka O., Itskovitz-Eldor J., Benvenisty N. (2001). Establishment of human embryonic stem cell-transfected clones carrying a marker for undifferentiated cells. Curr. Biol..

[37] Blum B., Bar-Nur O., Golan-Lev T., Benvenisty N. (2009). The anti-apoptotic gene survivin contributes to teratoma formation by human embryonic stem cells. Nat. Biotechnol..

[38] Rong Z., Fu X., Wang M., Xu Y. (2012). A scalable approach to prevent teratoma formation of human embryonic stem cells. J. Biol. Chem..

[39] Ricordi C., Lacy P.E., Finke E.H., Olack B.J., Scharp D.W. (1988). Automated method for isolation of human pancreatic islets. Diabetes.

[40] Nano R., Bosco D., Kerr-Conte J.A., Karlsson M., Charvier S., Melzi R., Ezzouaoui R., Mercalli A., Hwa A., Pattou F. (2015). Human islet distribution programme for basic research: Activity over the last 5 years. Diabetologia.

[41] Ravassard P., Hazhouz Y., Pechberty S., Bricout-Neveu E., Armanet M., Czernichow P., Scharfmann R. (2011). A genetically engineered human pancreatic β cell line exhibiting glucose-inducible insulin secretion. J. Clin. Investig..

