# Supplementary material for: Treating iPSC-Derived β Cells with an Anti-CD30 Antibody–Drug Conjugate Eliminates the Risk of Teratoma Development upon Transplantation
Source: Int J Mol Sci. 2022 Aug 26;23(17):9699. doi: 10.3390/ijms23179699 (PMC9456216; doi:10.3390/ijms23179699)
Supplement: Supplementary file 1 [file ijms-23-09699-s001.zip › ijms-1871421-supplementary.pdf]

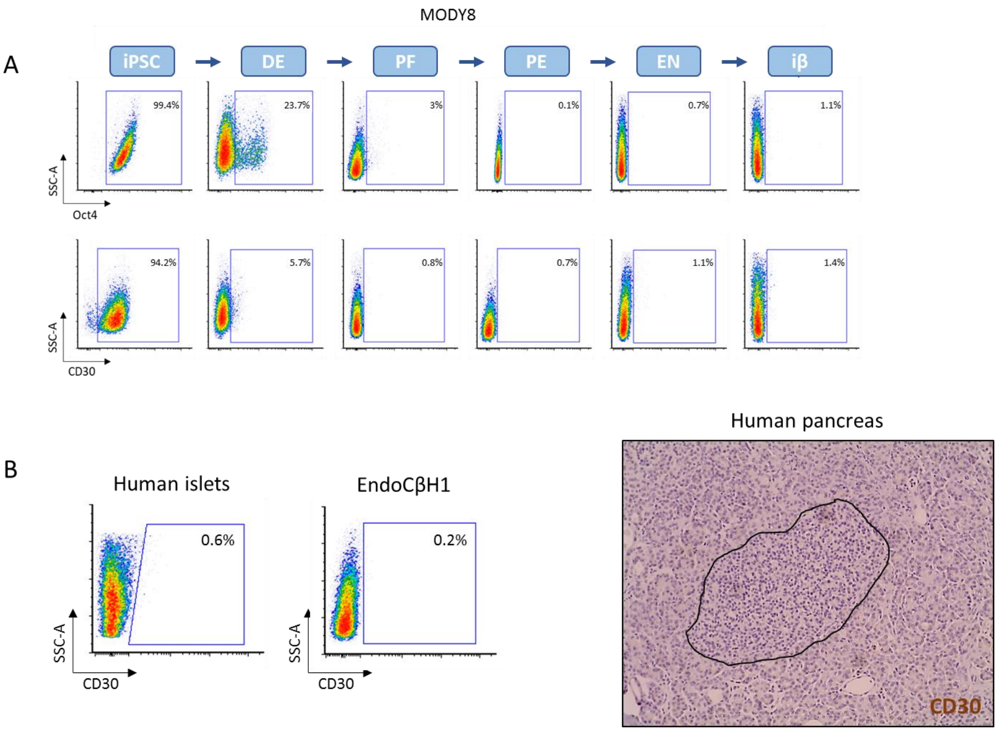

Figure S1. CD30 expression during iPSC differentiation and in human islets and EndoCβH1. (**A**). Representative plots of cells positive for Oct4 (upper panels) and CD30 (lower panels) in flow cytometry at the different steps of differentiation (iPSC, DE, PF, PE, EN, iβ) in MODY8-iPSC line. Gate delimitates positive cells. (**B**). On the left, representative dot plot of cells positive for CD30 in human islet from pancreas donor and immortalized β cell line; on the right CD30 staining on a section of cadaveric pancreas. Black line surrounds pancreatic islet.
